# HIV-specific cytolytic CD4 T-cell responses effectively control HIV infection in macrophages

**DOI:** 10.1186/1742-4690-9-S2-P274

**Published:** 2012-09-13

**Authors:** DZ Soghoian, M Flanders, K Sierra-Davidson, S Ranasinghe, S Cutler, I Davis, M Lindqvist, K Lane, B Kuhl, G Kranias, A Piechocka-Trocha, H Jessen, BD Walker, H Streeck

**Affiliations:** 1 Ragon Institute of MGH, MIT, and Harvard, Charlestown, MA, USA; 2 Praxis Jessen Jessen Stein, Berlin, Germany

## Background

HIV-specific cytolytic CD4 T-cell responses expand during acute HIV infection in individuals who control viremia and are associated with better disease outcome. Up to 75% of the HIV-specific CD4 T-cells exhibit a cytolytic phenotype during acute infection, but it is not understood how cytolytic CD4 T-cells contribute to viral control or what their primary target cells are.

## Methods

Using a novel, fluorescence-based single-round viral suppression assay, we assessed the ability of CD4 T-cells from HIV infected subjects to lyse infected macrophages. Elimination of infected macrophages and CD4 cytolytic phenotype were determined by flow cytometry. In addition, HIV-specific CD4 T-cell clones were generated and their cytolytic ability examined by Cr51-release and viral inhibition assays.

## Results

We observed significantly higher degranulatory HIV-specific CD4 T-cell responses in HIV controllers compared to progressors (p=0.015). Moreover, about 1/4 of all HIV-specific CD4 T-cell clones showed cytolytic activity by Cr51-release. Using a single-round viral suppression assay, we additionally observed that HIV-specific CD4 T-cells from chronically infected subjects were able to significantly lyse HIV infected macrophages (p=0.004). Elimination of HIV-infected macrophages was dose-dependent, up to 37% at E:T=5:1. Lytic ability could be observed ex vivo, and was enhanced after short term Gag-specific expansion in culture (11% to 32%, p=0.014). Furthermore, we observed that the inhibitory capacity of CD4 T cells could be abrogated using an HLA-DR blocking antibody. CD4 T-cell-mediated macrophage lysis was associated with strong HIV-specific cytolytic activity by intracellular cytokine staining and high expression of granzymes/perforin within HIV-specific CD4 T-cells.

## Conclusion

Our data demonstrate that HIV-specific CD4 T-cells derived from infected individuals have the ability to eliminate infected macrophages. These data suggest a role for HIV-specific cytolytic CD4 T-cell responses, in the absence of CD8 T cell responses, in the lysis of HIV-infected macrophages, which represent important reservoirs for viral infection and viral dissemination.

